# Mechanism of Remediation of Cadmium-Contaminated Soil With Low-Energy Plant Snapdragon

**DOI:** 10.3389/fchem.2020.00222

**Published:** 2020-04-08

**Authors:** Yang Zhi, Qixing Zhou, Xue Leng, Chunlei Zhao

**Affiliations:** ^1^School of Pharmaceutical Engineering, Shenyang Pharmaceutical University, Shenyang, China; ^2^Key Laboratory of Pollution Processes and Environmental Criteria (Ministry of Education)/Tianjin Key Laboratory of Environmental Remediation and Pollution Control, College of Environmental Science and Engineering, Nankai University, Tianjin, China; ^3^School of Traditional Chinese Medicine, Shenyang Pharmaceutical University, Shenyang, China

**Keywords:** snapdragon, low-energy plant, cadmium pollution, mineral nutrients, root exudates

## Abstract

In the process of remediation of contaminated soil, we should give full play to the role of low-energy plants and fully display the concept of modern energy-saving and environmental protection. Phytoremediation is an effective method to remediate cadmium-contaminated soil, and root exudates play an important part in this process. Here, the response of snapdragon in a pot-culture experiment under two concentrations of Cd (1.0 and 2.5 mg/kg) was evaluated. Snapdragon is a medicinal plant with low energy consumption, which has low requirements on environmental factors and strong resistance. The results showed that both Cd concentrations interfere with the uptake of B, P, Cu, Mn, Mo, and Zn by the soil. The results also showed that plant type and Cd stress can significantly change the concentrations and species of root exudates. The metabolic changes of root exudates revealed the active defense mechanism of plants to Cd stress: up-regulating of amino acids to sequester/exclude Cd, regulation of citric acid on chelation/complexation, and precipitation of cadmium ions. The application of snapdragon can effectively reduce energy consumption and gradually improve the utilization rate of vegetation, which promotes the degradation of cadmium pollutants in soil.

## Introduction

Low-energy-consuming plants can effectively photosynthesize to a certain extent, which has an impact on the surrounding vegetation, and can help all kinds of vegetation absorb more nutrients, so as to achieve the unity of economic and social benefits. Planting snapdragon does not need to use chemical fertilizers and pesticides; it needs to match the environment and can achieve minimum consumption. Snapdragon, a perennial herbaceous plant, is highly valued in traditional Chinese medicine. It provides treatments for clearing away heat, detoxicating, cooling blood, and relieving swelling. The application of snapdragon can accelerate the speed of photosynthesis and respiration of vegetation, and to some extent, it can effectively alleviate the environmental pressure of the city.

With the continuous development of social economy, industrial and agricultural activities, such as the application of fertilizers, lime, manure, sewage sludge, and compost, result in loss of energy and soil contamination by heavy metals (Zhou et al., 2016). In China, Cd has posed a serious threat to the safety of crops and food production; one-fifth of the farmland, at least 2 × 10^5^ km^2^ of farmland, has been polluted by Cd to varying degrees (Fan et al., 2013). Cadmium (Cd) is a potentially harmful heavy metal that can be toxic to plants even at very low concentrations (0.5 μg Cd g^−1^ soil). In recent years, the content of heavy metals in medicinal plants has attracted worldwide attention, because these elements usually enter the food chain through plants and gradually pass to the final consumers, resulting in a large number of health problems. Accumulation of cadmium in soils can lead to soil degradation and decreased yields of medicinal plants, with long-term risks to ecosystems and human health (Chary et al., 2008). It is important to focus on medicinal plants, especially the monitoring of Chinese medicinal materials from areas that may be polluted by heavy metals to prevent heavy metals from exceeding standards and ensure the safety of medication.

Conventional techniques to remediate heavy metal(loid)s from contaminated soils are based on physical, chemical, and biological methods, which may be used in combination with one another to remediate contaminated sites. Despite high efficiency, majority of these techniques are costly, have high energy consumption, and are environmentally destructive (Khalid et al., 2017). In order to reduce the toxicity of cadmium in medicinal plants, we screened the common medicinal plants and found that snapdragon was a low cadmium accumulation plant. Further, we would like to understand the restoration mechanism of snapdragon. Among them, root exudates, which are closely related to the roots of plants, is preferred because it can affect the growth and metal uptake of plants in an environmentally friendly way (Zhou et al., 2016; Li et al., 2018). Root exudates are mainly photosynthetic products transferred from the root and released to the rhizosphere (Walker et al., 2003) Plant roots can release a variety of compounds including carbohydrates, organic acids, amino acids, fatty acids, sterols, and vitamins (Carvalhais et al., 2011; Lu et al., 2017). These compounds play an important role in plant stress resistance and external removal of pollutants. A large amount of evidence shows that plants chelate or complex toxic metals (such as Al, Cd, Zn, Fe, and Cu) by up-regulating certain organic acids (including amino acids), thus hindering their transfer to aboveground plant tissues. It is well-known that about 30–40% of the photosynthate carbon will be transferred to the rhizosphere in the form of root exudates, including organic acids, amino acids, sugars, proteins, phenolic compounds, and CO_2_. In the soil–plant system, the interaction between organic acids and metals is of great significance for the dissolution/combination of metals in the insoluble mineral phases in soils.

Therefore, the specific objectives of this research were to (1) investigate the uptake, translocation, and accumulations of Cd in low-energy plant snapdragon tissues; (2) explore the response of root exudates (organic acids, amino acids) of snapdragon to cadmium toxicity; and (3) test the influence of Cd on the accumulation of mineral nutrient (Ca, Cu, Fe, Mg, Mn, Mo, P, and Zn) in different tissues of snapdragon.

## Materials and Methods

### Experimental Site and Soil Characterization

The pot-culture experiment was carried out in a greenhouse at the Shenyang Pharmaceutical University. The temperature in greenhouse was maintained at 27°C during the day and 21°C at night. Soil samples were collected from the surface soil of meadow burozem topsoil (0–20 cm) in a field at the Shenyang Experimental Ecology Station (41° 31′ N and 123° 41′ E), Chinese Academy of Sciences, which is located at the south of Shenyang City, Liaoning Province, China, where the mean temperature was about 6–10°C. The frost-free period lasts 127–164 days per year. According to the routine analysis methods of pesticides and soil, the basic physical and chemical properties of the tested soil were analyzed (Lu, 1999). Chemical analysis showed that soil organic matter content was 2.62%, pH value was 6.87, cation exchange capacity (CEC) was 0.473 mol kg^−1^, and Cd background concentration was 0.15 mg kg^−1^.

### Experimental Design

The soil sample was mixed with an appropriate concentration of CdCl_2_·2.5 H_2_O, ground and passed through a 4-mm mesh, and then 2.5 kg of soil was filled in each plastic basin (Φ = 20 cm, *H* = 15 cm). There were three treatments: CK (control, no Cd) and two Cd treatments: T_1_ (1.0 mg Cd kg^−1^ soil) and T_2_ (2.5 mg Cd kg^−1^ soil). Two levels (1.0 and 2.5 mg Cd kg^−1^ soil) indicate low to moderate pollution according to the single pollution index evaluation method and the soil grading scale for heavy metal contamination (Zhou and Song, 2004; Chai et al., 2006; Liu et al., 2010). Accurately weigh 500 g of the corresponding soil and put it into a 300-mesh nylon rhizosphere bag. The diameter of the rhizosphere bag was about 15 cm. One rhizosphere bag is placed in each plastic flowerpot, and the soil in the bag is used as the rhizosphere soil of the plant. The soil is watered and then covered with tarpaulin to keep it perfectly balanced for up to 8 weeks, a long enough time to allow a natural balance of the various adsorption mechanisms in the soil. These pots are arranged in a completely random square design. Each treatment was repeated three times to minimize experimental errors.

Snapdragon seeds used in the experiment came from a seed company in Shenyang, China. The seeds were sterilized with 2% (V/V) hydrogen peroxide for 10 min and then washed with distilled water for several times. Subsequently, the seeds are transferred to paper towels to germinate. Place the rolled paper towel with the seeds in a box with distilled water on the bottom and leave it in the dark for 3 days, then expose it to light for 1 day. After germination in the greenhouse, 6 seeds were sown in each pot. The pots did not use fertilizers. During the experiment, water loss was replenished to maintain 75% of the soil's water holding capacity, and a dish was placed under each pot to collect potential leachate.

### Cd and Nutrient Elements Analysis

At harvest time, snapdragon plants are thoroughly washed three times with tap water and then three times with deionized water. These plants are divided into roots and shoots. The samples were dried at 105°C for 5 min and oven-dried for 3 days at 70°C, then the rhizosphere soil was collected by shaking root method and put into the rhizosphere bag. Soil samples were dried, ground with a mortar and pestle, and passed through a 0.149-mm sieve (Wei and Zhou, 2004). Plant sample (0.50 g) and a soil sample (0.50 g) were digested with 12 ml of solution containing 87% concentrated HNO_3_ and 13% concentrated HClO_4_ (V/V) (Wei et al., 2005). The concentration of Cd, Ca, Cu, Fe, Mg, Mn, Mo, P, and Zn was analyzed using ICP-AES (Spectro arcos, Germany). The recoveries for all the elements were between 92 and 99%.

### Root Exudates Collection and Analysis

Weigh about 2 g of rhizosphere soil and put it into a 10-ml centrifuge tube. On the basis of the previous study, 4 ml of solution containing 0.1% H_3_PO_4_ was added to the solution of each component of root exudate to inhibit microbial activity. The tube was shaken on a rotary shaker at 200 rpm in the dark to achieve apparent equilibrium desorption. All microorganisms were then removed by centrifugation at 5,000 rpm for 5 min followed immediately by centrifugation with a syringe filter (0.45 μm). The organic acids in the soil was extracted and analyzed using a reported method (Sun et al., 2010). The soil samples (0.25 g) were digested with 6 mol/L HCl 20 ml at 105°C for 12 h in a 100-ml hydrolysis bottle. The amino acids in the soil were extracted and analyzed (Hou et al., 2006).

### Statistical Analysis

Data were analyzed by Excel 2018 and SPSS 18.0. All values are expressed as mean ± standard deviation (*n* = 3). When *P* < 0.05, the treatment effect was statistically significant. Data were analyzed by one-way ANOVA and Tukey multiple range test. All results are expressed as dry weight.

## Results and Discussion

### Impact of Cd on Snapdragon Growth

**Figure 1:**
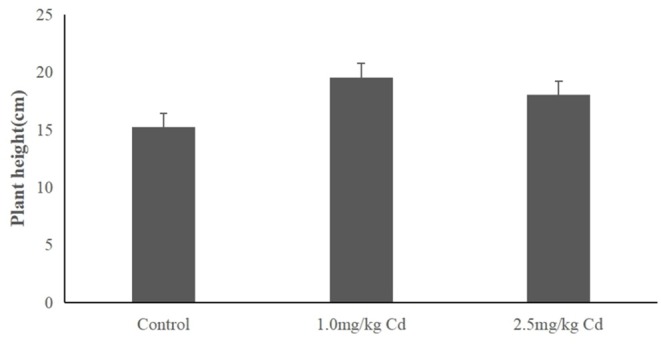
The height of snapdragons in the pot-culture experiment.

The plant height of snapdragon in different Cd treatments was depicted in Figure 1. Snapdragon plants exhibited significant increase in plant height compared to the control after 60 days of exposure to 1.0 and 2.5 mg/kg of Cd. The result indicated that the snapdragon plants had tolerance to Cd toxicity.

**Figure 2:**
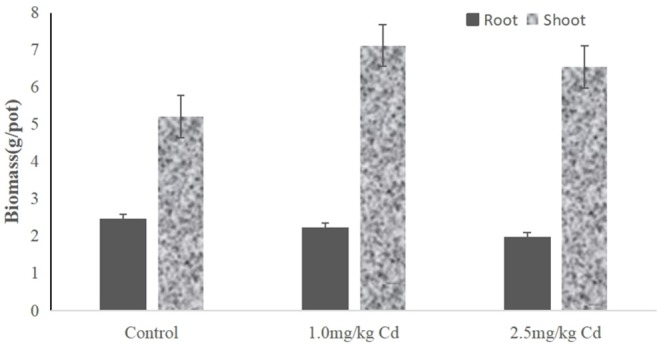
The biomass of different tissues from snapdragons treated with Cd at 0, 1.0, and 2.5 mg/kg.

Root biomass decreased by 9.8 and 11.3% at 1.0 and 2.5 mg/kg Cd treatment, respectively (Figure 2), but only at 2.5 mg/kg was Cd treatment significant (*P* < 0.05). Compared with the root system, the effect of Cd on the aboveground biomass (including stem and leaf) was not significant. The shoot biomass of the snapdragon plants under 1.0 mg/kg Cd also increased significantly (*P* < 0.05) compared with control and Cd at 2.5 mg/kg. A large number of studies have shown that cadmium has a certain dose effect on plant growth; that is, low concentration of cadmium has a stimulating effect on plant growth (Jia et al., 2013; Zhou et al., 2013; Janota et al., 2015; Guo et al., 2018). Similar results have been reported in asparagus bean (Zhu et al., 2007), paddy rice (Yu et al., 2006), Chinese cabbage (Liu et al., 2010), tomato (Zhu et al., 2006), and soybean (Zhi et al., 2014). Two possible reasons can be suggested. One possible reason is that metal ions can act as activators of enzymes in cytokinin metabolism and promote plant growth (Shentu et al., 2008). Another possible reason is that low concentrations of cadmium hyperpolarize the plasma membrane at the root surface, increasing the transmembrane potential as a source of energy for cation absorption (Kennedy and Gonsalves, 1987). Therefore, it is difficult for farmers to find cadmium pollution in snapdragon, which will increase the harm of cadmium pollution products in snapdragon to human health.

### Cd Accumulation and Distribution

**Figure 3:**
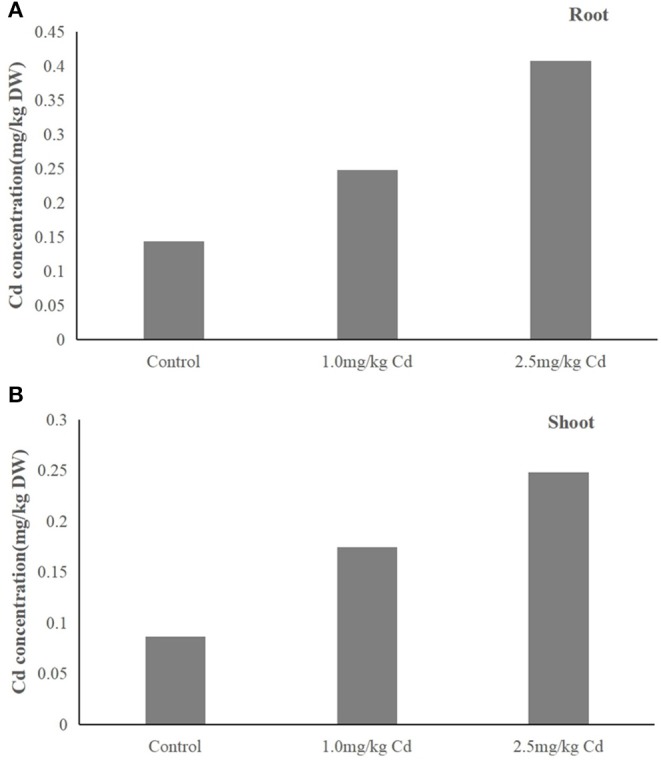
**(A,B)** Distribution of Cd in the tissues of snapdragons under two Cd treatments.

We observed that Cd concentration in plants (root and shoot) treated with 1.0 and 2.5 mg/kg was significantly higher than that of control (Figure 3). The results showed that Cd content in plants increased with the increase of Cd concentration in soil. Cd was absorbed by plants and transported from roots to shoots within 60 days. There was no significant (*P* > 0.05) difference in distribution of Cd in the tissue of the plant under two treatments. Cadmium was mainly in roots (55.3–58.6%) and then in shoots (41.4–44.7%). Therefore, most of the Cd absorbed by the plant is reused in the root chamber, and the rest of the Cd is transported to the upper tissue. Cd is transported to the upper tissue. This is consistent with previous reports showing that the Cd is located primarily at the root. Jarvis et al. (1976) reported that more than 70% of supplied Cd was incorporated in roots of *Zea mays* and other plants (Jarvis et al., 1976).

The accumulation of cadmium in plants is closely related to the physiological processes of plant absorption, transportation, and detoxification. It is generally accepted that the uptake of heavy metals in soil is either passive, that is, a large amount of water enters the roots, or active transport through the plasma membrane of root epidermal cells (Yoon et al., 2006). The distribution of Cd in the aboveground and underground parts of plants is affected by the absorption, transportation, chelation, and compartmentalization of Cd (Wei et al., 2012).

### EF and TF

Enrichment factors (EF) (Chen et al., 2004; Zhi et al., 2014) were calculated using the following formula to assess the ability of plants to accumulate heavy metals:

$$\begin{array}{l} \text{EF}=\frac{C_{shoot}}{C_{soil}} \end{array}$$

where *C*_*shoot*_ is the average Cd concentration (DW) of the plant and *C*_*soil*_is the total concentration of Cd in the corresponding soil. The EF values in plants were found to be lower than 0.20 under the two Cd treatments (Table 1).

**Table 1:** Enrichment Factor (EF) and Translocation Factor (TF) in snapdragons under different cadmium concentrations.

	**TF**	**EF**
Control	0.60	
1.0 mg/kg Cd	0.71	0.17
2.5 mg/kg Cd	0.81	0.10

In addition, translocation factor (TF) is the ratio of metal concentrations in the shoot to those in the root (Bose and Bhattacharyya, 2008). By calculating transfer factors (Baker and Whiting, 2002; Zhi et al., 2014), we assessed the potential for Cd transfer from root to shoot as follows:

$$\begin{array}{l} \text{TF}=\frac{C_{shoot}}{C_{root}} \end{array}$$

where *C*_*shoot*_ is the average Cd concentration (DW) of the plant and *C*_*root*_ is the average Cd concentration (DW) of the root of each corresponding plant. There was an upward tendency that all the TF values were lower than 1.0 (Table 1). It is worth noting that with the increase of Cd concentration, the TF value tends to increase, while the EF value decreases (Table 1).

The results showed that the uptake capacity of Cd in soil was low, but the transport capacity of root to shoot was relatively high. In snapdragon, when the Cd concentration in soil increased slightly, the Cd concentration in shoot increased correspondingly. It is considered that the excessive migration of Cd from soil to plants can make up for the deficiency of Cd migration from roots to shoots, which can easily lead to the risk of Cd pollution in snapdragon. Therefore, pollution-safe varieties are varieties that grow in contaminated soil and have concentrations of certain pollutants in their edible parts that are low enough to be safe to eat, which is a cost-effective way to reduce cadmium accumulation in crops (Liu et al., 2005; Yu et al., 2006). Based on previous investigations (Baker and Whiting, 2002; Zhi et al., 2015), four criteria were applied to screen for medicinal plants with low cadmium accumulation: (1) the edible fraction should have a Cd concentration <0.3 mg/kg DW according to Green Standards of Medicinal Plants and Preparations for Foreign Trade and Economy (WM/T2-2004); (2) EF <1.0; (3) TF <1.0; and (4) Cd tolerance as measured by shoot biomass and height when grown in contaminated soil. The results showed that snapdragons were a low Cd accumulator when the concentration of Cd was <1 and 2.5 mg/kg. In addition, the EF and TF of these plants were lower than 1.0.

### Mineral Nutrients

Cadmium is a non-essential element (Liñero et al., 2015), which can actively enter plant cells through the absorption mechanism and absorb essential elements such as Zn, Ca, and Fe (Lu et al., 2009). As shown in Table 2, there were significant differences in nutrient elements among the different Cd treatments. The concentrations of B, P, Fe, Ca, Cu, and Mg in different tissues of snapdragon decreased. Except for Cu in root (*p* = 0.071) and Mg in shoot (*p* = 0.114), the others decreased significantly (*p* ≤ 0.05). In addition, Cd reduced the uptake of Mn by roots, but had little effect on shoots. Several studies have shown that excessive Cd can reduce the absorption of minerals. The addition of high concentrations of Cd to the soil reduced the uptake of Mg by roots and the accumulation of Mg in brown rice (Khaliq et al., 2019). Carvalho et al. also found that the avoidance of leaf B accumulation reduced the response of tomato to Cd toxicity. The degree of Cd toxicity was enhanced by both the excess in leaves and Mn deficiency in roots (Carvalho et al., 2019). Cd is believed to have a common entry route with Fe and Mn (Ishimaru et al., 2012; Sasaki et al., 2012)).

**Table 2:** Effects of Cd on mineral nutrient accumulation in snapdragon tissues (mg/kg, DW).

	**Zn**	**B**	**P**	**Fe**	**Mn**	**Ca**	**Cu**	**Mo**	**Mg**
**Root**									
Control	386^a^	85^a^	211^a^	510^a^	168^a^	622^a^	25^a^	119^a^	6,593^a^
1.0 mg/kg	355^ab^	59^ab^	190^b^	263^ab^	134^ab^	621^a^	12^a^	110^b^	5,197^b^
2.5 mg/kg	560^b^	47^b^	152^b^	211^b^	115^b^	617^b^	11^a^	112^b^	3,341^c^
*p*-value	0.021	0.03	0.009	0.041	0.004	0	0.071	0.001	0.033
**Shoot**									
Control	17^a^	58^a^	145^a^	28^a^	32^a^	379^a^	11^a^	39^a^	1,341^a^
1.0 mg/kg	13^ab^	36^b^	129^b^	16^b^	20^ab^	314^ab^	7^b^	27^b^	689^a^
2.5 mg/kg	28^b^	35^b^	118^b^	12^b^	28^b^	192^b^	5^b^	28^b^	341^a^
*p*-value	0.047	0.029	0.001	0.031	0.017	0.033	0.049	0.015	0.114

*Different letters stand for statistical differences at p ≤ 0.05*.

The interaction between Cd and Zn was antagonistic at low concentration of Cd, but synergistic at high concentration of Cd. Under the treatment of 2.5 mg/kg Cd, the absorption and accumulation of Zn in roots and shoots increased. Under the treatment of 2.5 mg/kg Cd, the uptake and accumulation of Zn in roots and shoots increased. Lasat et al. (2000) discovered the Zn transporter gene *ZNT1*, which can promote the transport of Cd in plants. The long-distance transport mechanism of Cd in plants may be the same as that of Zn (Liu et al., 2003). The content of Fe in different parts of snapdragon was significantly negatively correlated with the concentration of Cd, which was consistent with previous studies (Sasaki et al., 2012; Khaliq et al., 2019). An Fe-Cd correlation was found in the roots and shoots of snapdragon, as both Fe and Cd are transported by same transporters (*OsNRAMP1*) (Curie et al., 2000). It is clear that addition of high concentrations of Cd inhibited Fe accumulation in the snapdragon. Therefore, the accumulation of Fe in snapdragon was inhibited by adding high concentration of Cd.

The decrease of Ca concentration in various tissues is due to the fact that Cd and Ca share a common transport system. Studies have shown that a large number of Cd in plants do not enter the symplast, but remain in the cell wall. Beyersmann and Hechtenberg proved that Cd could interfere with the activation of some signal transduction pathways based on the binding characteristics of Cd to some calcium binding sites in animal cells. This leads to a decrease in the amount of Ca accumulated by plants (Beyersmann and Hechtenberg, 1997).

### Metabolomics Analysis of Amino Acids Extracted From Root

**Figure 4:**
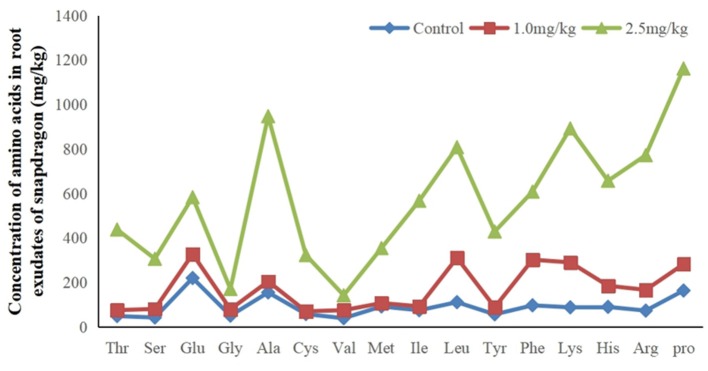
Sixteen up-regulated amino acids in root exudates in response to exposure at 1.0 and 2.5 mg/kg Cd.

The research results listed in Figure 4 indicate that there were 16 amino acids to be detected in root exudates. They are Alanine (Ala), Arginine (Arg), Cysteine (Cys), Glutamic acid (Glu), Glycine (Gly), Histidine (His), Isoline (Ile), Leucine (Leu), Lysine (Lys), Methionine (Met), Phenylalanine (Phe), Proline (Pro), Serine (Ser), Threonine (Thr), Tyrosine (Tyr), and Valine (Val). They were significantly up-regulated in response to Cd. Increased amino acid secretion may be an active defense response in snapdragon. When plants are under stress, root exudates can change the form of insoluble nutrients in soil to facilitate plant absorption and regulate rhizosphere microbial activity, which is one of the mechanisms of plants coping with stress. Amino acid is an important component of soil organic nitrogen and an important source of nutrients for microorganisms in soil. Amino acids are consumed by microorganisms to synthesize growth regulators, which regulate and stimulate plant growth. The metabolic products of microorganisms will affect the secretion of amino acids. Up-regulated amino acids can provide multiple binding sites for Cd, which hinders Cd transport from the root cell membrane. Therefore, on the one hand, the amino acids secreted by the root of snapdragon under cadmium stress may form a stable chelate complex with cadmium ion and reduce its activity. On the other hand, it may indirectly participate in the process of plant resistance to cadmium toxicity by affecting the species, quantity, and physiological activity of rhizosphere microorganisms. This mechanism needs further discussion and demonstration.

Previous studies have shown that amino acids play an important role in the process of chelating Cd^2+^. Tang (1998) observed that amino acid could reduce the toxicity of metal ions. Up-regulated amino acids may also reflect an attempt by snapdragon to quarantine cadmium in the stem. Amino acids may also be secreted in xylem sap and bound to Cd^2+^ in transpiration. Amino acids in root exudates not only can bind metals but also can act as signal molecules and have antioxidant defense function. These results suggest that amino acids may detoxify cadmium by binding to cadmium ions.

### Detoxification of Organic Acids Secreted by Roots Under Cadmium Stress

**Figure 5:**
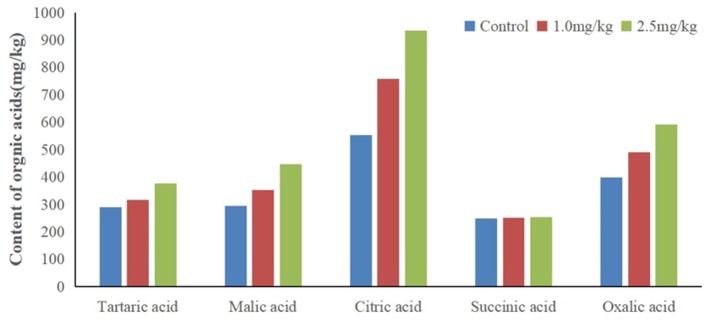
Contents of different organic acids in root exudates of snapdragons under cadmium stress.

Organic acids can decrease the pH value of rhizosphere soil, activate insoluble minerals in soil, and improve the bioavailability of heavy metals. In this study, snapdragon root exudates were collected, and the organic acids were identified by HPLC (Figure 5). Tartaric acid, citric acid, succinic acid, malic acid, and oxalic acid were found in snapdragon root exudates. Surprisingly, patterns of succinic acid were not changed by Cd, which indicates that succinic acid did not respond to Cd stress. In contrast, tartaric acid, citric acid, malic acid, and oxalic acid were up-regulated by Cd. The excretion of citric acid was the largest, and the percentage of citric acid in organic acid increased with the increase of cadmium stress concentration, and exceeded 40% under two cadmium stress concentration. The citric acid content in the root exudate of snapdragon treated with 1.0 and 2.5 mg/kg Cd was 1.1 times and 1.4 times higher than that of the control, respectively. It is known that organic acids have strong binding capacity with heavy metals. Hoffland et al. considered that the abundance of citric acid in rhizosphere can be one factor contributing to the poor efficiency of P amendment practices (Hoffland et al., 1992). These organic acid radical ions can form metal cation–organic acid complexes with metal ions such as iron through complexation/chelation, which can promote the release of phosphorus in insoluble phosphorus compounds and alleviate the shortage of available phosphorus in soil. Previous studies have shown that citric acid can improve the solubility of ferrous iron, but it is reluctant to provide iron to Caco-2 epithelial cells, resulting in iron malabsorption (Salovaara et al., 2003). The action of citric acid may occur through its carboxyl and hydroxyl groups, which prevent the polymerization of iron hydroxides by forming soluble complexes with iron (Ballot et al., 1987). Therefore, the detoxification mechanism of snapdragon on cadmium stress is to release organic acids to combine with cadmium ion or to change the existing form of cadmium ion in order to reduce the toxicity of cadmium on plant roots. Other reports showed that organic acids, especially those taking part in the tricarboxylic acid cycle, could facilitate mineral nutrient uptake and sequester toxic metals. Zinc plays an important role in photosynthesis, respiration, active oxygen metabolism, and interaction between phosphorus and zinc in plants. Under zinc deficiency stress, the transportation and utilization of zinc in plants can be affected by increasing the concentration of organic acids, which can act as an important ligand binding to zinc in plants and participate in the transportation, distribution, and detoxification of zinc in plants (Rehman et al., 2012). Citric acid has shown to aid in the absorption of zinc by snapdragons. The up-regulation of citric acid may be an active process to increase the solubility, absorption, and transport of Cd in snapdragon. Because citric acid can form a stable extracellular complex with Cd, it can reduce the transfer of Cd from roots to shoots.

We found that the pH went from 6.57 (1.0 mg/kg Cd) to 6.27 (2.5 mg/kg Cd). The root exudates decreased the pH value of rhizosphere soil by 0.3–0.6 pH units. The decrease of pH value resulted in the higher concentration of soluble Cd^2+^ than that of the control. The results showed that citric acid had a strong capacity to dissolve Cd by decreasing the pH of the system. The roots of some plants, such as wheat and buckwheat, excrete organic acids (e.g., oxalic, malic, and citric) that bind Cd^2+^ and prevent it from entering the roots (Dong et al., 2007). Up-regulation of amino acids and citric acid may be a strategy for plants to inhibit Cd absorption and remove Cd toxicity.

## Conclusions

The application of snapdragon can effectively reduce energy consumption and gradually improve the utilization rate of vegetation, which reflects the concept of energy saving and environmental protection to a certain extent and meets the actual needs of low-carbon life. The results showed that both Cd concentrations interfere with the uptake of B, P, Cu, Mn, Mo, and Zn by the soil. Root exudates can affect the absorption of heavy metals by changing the physical and chemical properties of rhizosphere soil. Because the nutrient source of rhizosphere microorganisms comes from root exudates, the changes of amino acids, carboxylic acids, and carbohydrate metabolites may affect the activity and community of rhizosphere microorganisms. The results showed that Cd changed the content of amino acids and organic acids in root exudates and thus changed the feedback relationship between plants and rhizosphere microorganisms. These findings are of great significance for understanding the chemical behavior of heavy metals at the root/soil interface and reducing the toxicity of heavy metals to medicinal plants.

## Data Availability Statement

All datasets generated for this study are included in the article/supplementary material.

## Author Contributions

YZ conceived and set up the experiment, analyzed the results, and wrote the manuscript. QZ conducted the experiment, analyzed the results, and helped YZ write the manuscript. XL and CZ analyzed the results and helped YZ write the manuscript. All authors reviewed the manuscript.

### Conflict of Interest

The authors declare that the research was conducted in the absence of any commercial or financial relationships that could be construed as a potential conflict of interest.
